# Sugar versus fat: elimination of glycogen storage improves lipid accumulation in *Yarrowia lipolytica*

**DOI:** 10.1093/femsyr/fox020

**Published:** 2017-04-17

**Authors:** Govindprasad Bhutada, Martin Kavšček, Rodrigo Ledesma-Amaro, Stéphane Thomas, Gerald N. Rechberger, Jean-Marc Nicaud, Klaus Natter

**Affiliations:** 1 Institute of Molecular Biosciences, NAWI Graz, University of Graz, Humboldtstrasse 50/II, 8010 Graz, Austria; 2 Micalis Institute, INRA, AgroParisTech, Université Paris-Saclay, 78350 Jouy-en-Josas, France; 3 Omics Center Graz, BioTechMed Graz, 8010 Graz, Austria

**Keywords:** oleaginous yeast, triacylglycerol, glycogen synthase, storage metabolism

## Abstract

Triacylglycerol (TAG) and glycogen are the two major metabolites for carbon storage in most eukaryotic organisms. We investigated the glycogen metabolism of the oleaginous *Yarrowia lipolytica* and found that this yeast accumulates up to 16% glycogen in its biomass. Assuming that elimination of glycogen synthesis would result in an improvement of lipid accumulation, we characterized and deleted the single gene coding for glycogen synthase, *YlGSY1*. The mutant was grown under lipogenic conditions with glucose and glycerol as substrates and we obtained up to 60% improvement in TAG accumulation compared to the wild-type strain. Additionally, *YlGSY1* was deleted in a background that was already engineered for high lipid accumulation. In this obese background, TAG accumulation was also further increased. The highest lipid content of 52% was found after 3 days of cultivation in nitrogen-limited glycerol medium. Furthermore, we constructed mutants of *Y. lipolytica* and *Saccharomyces cerevisiae* that are deleted for both glycogen and TAG synthesis, demonstrating that the ability to store carbon is not essential. Overall, this work showed that glycogen synthesis is a competing pathway for TAG accumulation in oleaginous yeasts and that deletion of the glycogen synthase has beneficial effects on neutral lipid storage.

## Teaser

Glycogen and neutral lipid synthesis are competing pathways and the elimination of glycogen storage results in improved lipid synthesis in the yeast *Yarrowia lipolytica*.

## INTRODUCTION

*Yarrowia lipolytica* is a respiratory oleaginous yeast mainly found in dairy and meat products, as well as in polluted sea water. It has been widely studied for its ability to degrade hydrocarbons and for production of citric acid and single cell proteins (Nicaud 2012). In addition, its natural capability to store high amounts of neutral lipids (NLs) in the form of triacylglycerol (TAG) is investigated because it can serve as a sustainable feedstock for biodiesel production or for the biosynthesis of fatty acids (FA) (Ledesma-Amaro and Nicaud 2016) and fine chemicals (Abghari and Chen 2014). Besides TAG, glycogen serves as a second storage form for excess carbon. Microorganisms continuously sense the nutritional status of their environment and adapt their growth and metabolism to changing conditions. The accumulation of carbon stores in the form of glycogen and/or TAG is regarded as a strategy to deal with extended periods of starvation or other unfavorable conditions. Glycogen metabolism is highly conserved from yeast to humans. In baker's yeast, glycogen accumulates at the onset of stationary phase and can be strongly induced by stress conditions such as a limitation for nitrogen, sulfur or phosphorous when glucose is available (François and Parrou 2001). A similar behavior is found in bacteria (Preiss and Romeo 1994). Furthermore, Parrou *et al.* (1999) found changes in glycogen and trehalose accumulation as a response to the gradual depletion of nutrients from carbon or nitrogen-limited media. Under glucose limitation, they observed glycogen accumulation after the late logarithmic phase, whereas under nitrogen limitation, accumulation of glycogen was only observed after complete depletion of the nitrogen source. For oleaginous yeasts such as *Apiotrichum curvatum* and *Rhodosporidium toruloides*, it was shown that under nitrogen limitation glycogen can account for 10%–45% of the total biomass (Evans and Ratledge 1984; Park, Murphy and Glatz 1990). Similar results were found in a recent study that investigated the potential of several yeasts for FA production (Lamers *et al.*^2016^).

Like glycogen accumulation, NL synthesis and storage is greatly induced in response to nitrogen limitation. Indeed, a high ratio of carbon to nitrogen is the condition that is typically used for oleaginous yeasts to obtain high yields of TAG. However, there is only limited knowledge about factors that co-regulate the accumulation or mobilization of both glycogen and NL pools. Upon glucose depletion, the AMPK homolog Snf1p promotes cellular processes such as respiration, glycogen accumulation, peroxisome biogenesis and aging, and downregulates anabolic pathways such as amino acid and *de novo* FA synthesis (Conrad *et al.*^2014^). Under glucose repression, the transcriptional repressor Mig1p binds to the promoter of *GSY2*, the gene coding for the major glycogen synthase activity, and inactivates glycogen synthesis, whereas Snf1p phosphorylation of Mig1p releases this repression and promotes glycogen accumulation (Hardy, Huang and Roach 1994; Wang *et al.*^2001^). The anabolic process of FA synthesis is also subject to regulation through Snf1p, which catalyzes the phosphorylation of the acetyl-CoA carboxylase Acc1p, resulting in the inactivation of the first committed step in FA synthesis (Woods *et al.*^1994^). However, it has to be assumed that Snf1p is not the only regulator of storage metabolism because its deletion results in opposite effects for the two storage pools, a decrease in glycogen and an increase in NLs (Wilson, Wang and Roach 2002; Bozaquel-Morais *et al*. 2010).

Glycogen metabolism in *Y. lipolytica* has not been investigated in detail yet. Queiroz-Claret *et al. (*2000) reported a 76-kDa monomeric protein as a putative glycogen synthase. Contrary to *Saccharomyces cerevisiae, Y. lipolytica* was reported to show glycogen synthase activity already during the exponential phase of growth. This activity was increasing synchronously with the increase in activities of protein phosphatase 2A (PP2A), whereas, with the onset of stationary phase, protein kinase CK2 activity increases and phosphorylation of glycogen synthase results in depletion of glycogen pools. Recently, Dulermo *et al.* (2015) also showed that *Y. lipolytica* accumulates 9% glycogen in the biomass under nitrogen-limiting conditions.

In this work, we characterized the glycogen synthase of *Y. lipolytica* and investigated the effects of a deletion of the encoding gene, *YALI0F18502g*. We show that this deletion results in a significant increase in NL accumulation, suggesting that the cellular carbon flux is redirected from glycogen to TAG synthesis. This effect was also observed in a strain that has already been genetically engineered for high lipid accumulation. Finally, since storage metabolism is assumed to support viability in stationary-phase cells, we studied the impact on chronological life span for a strain deleted for glycogen synthesis as well as for a strain that is deficient in both glycogen and TAG synthesis.

## MATERIALS AND METHODS

### Strains, media and cultivation conditions

All strains used in this study are listed in Table 1. Media and growth conditions for *Escherichia coli* and *Yarrowia lipolytica* have been described by Sambrook and Russell (2001) and Barth and Gaillardin (1996), respectively. Yeast cultures were grown in minimal media, consisting of the following components: 5 g L^−1^ (carbon limited) or 0.4 g L^−1^ (nitrogen limited) (NH_4_)_2_SO_4_; 3 g L^−1^ KH_2_PO_4_; 0.5 g L^−1^ MgSO_4_.7H_2_O; buffered at pH 5.7 with 2-(N-morpholino)ethanesulfonic acid (MES). The carbon sources, glucose or glycerol, were autoclaved separately and 1 mL L^−1^ sterile-filtered trace metal and 1 mL L^−1^ vitamin solution as described by Hong and Nielsen (2013) were added after autoclaving. Depending on the nitrogen concentration, we will refer to shake flask cultivations as carbon limited (C-lim: 5 g L^−1^ glucose or glycerol and 5 g L^−1^ ammonium sulfate) or nitrogen limited (N-lim: 20 g L^−1^ glucose or glycerol and 0.4 g L^−1^ ammonium sulfate). For cultivation of *Saccharomyces cerevisiae* strains, the C-lim and N-lim media contained 20 g L^−1^ glucose as carbon source. In C-lim cultivations, this glucose concentration results in approximately the same final biomass for baker's yeast as the concentration of 5 g L^−1^ for *Y. lipolytica*. Media for the growth of the NL-deficient mutants were supplemented with amino acids.

**Table 1.** Strains used in this study.

Strains	Genotype	Source
***Yarrowia lipolytica***		
*YlWT* (H222)	*MATa wild-type*	Barth and Gaillardin (1996)
*JMY322*	*MATa ura3-41*	Mauersberger *et al.* (2001)
*Ylgsy1Δ*	*MATa ura3-41 gsy1Δ::URA3*	This work
*YlTEFGSY1*	*MATa ura3-41 TEF^P^-GSY1*	This work
*obese* (JMY6210)	*MATa ura3-302 leu2-270 xpr2-322 tgl4Δ TEF^P^-GPD1 TEF^P^-DGA2-LEU2 URA3*	This work
*obese gsy1Δ* (JMY6212)	*MATa ura3-302 leu2-270 xpr2-322 tgl4Δ TEF^P^-GPD1 TEF^P^-DGA2-LEU2 gsy1Δ::URA3*	This work
*YlQM* (JMY1877)	*MATa ura3-302 leu2-270 dga1Δ lro1Δ are1Δ dga2Δ*	Beopoulos *et al.* (2012)
*YlPM*	*MATa ura3-302 leu2-270 dga1Δ lro1Δ are1Δ dga2Δ gsy1Δ*	This work
*JMY195*	*PO1d strain - MATa ura3-302 leu2-270 xpr2-322*	Barth and Gaillardin (1996)
*JMYgsy1Δ*	*PO1d strain - MATa ura3-302 leu2-270 xpr2-322 gsy1Δ*	This work

***Saccharomyces cerevisiae***		
*ScWT* (CEN.PK113-5D)	*MATa SUC2 MAL2-8^c^ ura3Δ*	Entian and Koetter (2007)
*gsy1Δgsy2Δ*	*MATa SUC2 MAL2-8^c^ gsy1Δ::loxP gsy2Δ::loxP*	This work
*gsy1Δgsy2Δura3Δ*	*MATa SUC2 MAL2-8^c^ gsy1Δ::loxP gsy2Δ::loxP ura3Δ::loxP*	This work
*ScWT* (BY4742)	*MATα his3Δ1 leu2Δ0 lys2Δ0 ura3Δ0*	Brachmann *et al.* (1998)
*ScQM* (YJP1078)	*MATα his3Δ1 leu2Δ0 lys2Δ0 ura3Δ0 ycr048wΔ::KanMX4 ynr019wΔ::KanMX4 yor245cΔ::KanMX4 ynr008wΔ::KanMX4*	Petschnigg *et al.* (2009)
*ScHM*	*MATα his3Δ1 leu2Δ0 lys2Δ0 ura3Δ0 ycr048wΔ::KanMX4 ynr019wΔ::KanMX4 yor245cΔ::KanMX4 ynr008wΔ::KanMX4*	This work
	*gsy1Δ gsy2Δ*	

Precultures were prepared in 50 mL of the same medium as used for main cultures and cultivated at 28°C on a rotary shaker at 180 rpm for 18–24 h. Prior to inoculation into the cultivation flask, the preculture was washed twice with deionized water to remove any residual media components from the culture. Shake flask cultivations were performed in round bottom 1 L flasks with 200 mL of medium. The shake flasks were inoculated from precultures to a starting OD_600_ of 0.05 and incubated at 28°C on a rotary shaker at 180 rpm for 72 h.

For the determination of extracellular metabolites, biomass, glycogen and lipid content samples from C-lim media were harvested in exponential phase and in stationary phase, and from N-lim media after 24, 48 and 72 h of cultivation.

For the determination of survival in stationary phase, cultures were grown in C-lim medium containing 5 g L^−1^ glucose. Diluted aliquots were plated onto YPD plates (1% yeast extract, 2% peptone, 2% glucose, 2% agar) to determine viability of the cultures. The data for the analysis of chronological lifespan are derived from two independent experiments.

### Molecular and genetic work

All PCR reactions were performed using Herculase II Fusion DNA Polymerase (Agilent Technologies Österreich GmbH, Vienna, Austria) or Pyrobest (Takara Bio Europe, Saint-Germain-en-Laye, France) for cloning and amplification of sequencing templates, and with Solis BioDyne FIREPOL DNA Polymerase (Medibena, Vienna, Austria) or GoTaq (Promega, Madison, WI, USA) for confirmation of chromosomal integration of the transformation cassettes. The restriction enzymes used in this study were obtained from Roche (Roche Austria GmbH, Vienna, Austria) or OZYME (Ozyme, Montigny-le-Bretonneux, France). The DNA fragments from PCR and restriction digestion were recovered from agarose gels using GeneJET kits (Thermo Fisher Scientific) or QIAgen Purification Kit (Qiagen, Hilden, Germany). Standard protocols for lithium acetate transformations were used for both *S. cerevisiae* (Gietz and Woods 2002) and *Y. lipolytica* (Le Dall, Nicaud and Gaillardin 1994). All primers are listed in Table S1 (Supporting Information).

### Overexpression of *YALI0F18502g*

Expression in a glycogen-deficient strain of *S. cerevisiae*, a *loxP* flanked *KanMX4* cassette from pYGFPgN (Prein, Natter and Kohlwein 2000), was amplified with the primers gsy1_del_f and gsy1_del_r, bearing overhangs for homologous recombination at the *GSY1* locus and resulting in the replacement of the *GSY1* ORF with the *KanMX4* cassette after transformation of the wild-type strain CEN.PK 113–7D. To excise the cassette after confirmation of the deletion of *GSY1*, the *URA3* marker of pSH47 (Güldener *et al.*^1996^) was exchanged with the nourseothricin resistance gene. The resulting plasmid, pSH47-NAT, was used to transform the *gsy1* deletion strain. After incubation of the transformants in galactose medium for expression of the *Cre* recombinase, the colonies that had lost the G418 resistance were selected and the excision of the *KanMX4* cassette was confirmed by control PCR. The same procedure was used to delete *GSY2* and *URA3*, resulting in a uracil auxotrophic *gsy1::loxP gsy2::loxP* mutant.

For the strong constitutive expression of *YALI0F18502g* in *S. cerevisiae*, the plasmid pYES2 (Thermo Scientific) was digested with *Swa*I and *Sph*I to excise the *f1* origin and the *GAL*1 promoter. The *TEF1* promoter, amplified with the primers TEF-fwd and TEF-rev from *S. cerevisiae* genomic DNA, was digested with *Sma*I and *Sph*I and ligated to the linearized vector, resulting in the plasmid pHEY-1. *YALI0F18502g* was amplified from genomic DNA of *Y. lipolytica* using the primers Yl_GSY_F/ Yl_GSY_R. The PCR product was digested with *Eco*RI and *Xba*I and ligated with the *EcoR*I/*Xba*I digested pHEY-1, resulting in pHEY-1/Yl*GSY1*. Successful cloning was confirmed by sequencing, and the vector was used to transform the glycogen-deficient *S. cerevisiae* strain.

Overexpression in *Y. lipolytica*: for the strong constitutive expression of *YALI0F18502g* in *Y. lipolytica*, the *YlTEF1* promoter was amplified by PCR from *Y. lipolytica* genomic DNA with the primers YlTEF1_GSYoe_F/ YlTEF1_GSYoe_R and circularized with the *Cla*I digested plasmid pGMKGSY_12 (see below) by Gibson assembly (Gibson *et al.*^2009^). This intermediate plasmid was again linearized with *Cla*I enzyme. The reading frame of the gene *YALI0F18502g* was amplified with the primers GSYoe_F/ GSYoe_R, and the linearized plasmid and the PCR product were assembled by Gibson assembly to pGMKTEFGSY_02, which was used for transformation of the *Y. lipolytica* strain deleted for *YALI0F18502g* (see below).

### Deletion of the glycogen synthase gene

The plasmid pFA6a-GFP(S65T)-KanMX6 (Wach *et al.*^1997^) was digested with *Not*I to excise the *GFP* and *KanMX6* coding regions. To generate a *URA3* cassette flanked by *loxP* sites, *URA3* together with its promoter and terminator was amplified from *Y. lipolytica* genomic DNA with the primers Yl_URA3_F1/Yl_URA3_R1, bearing *loxP* sites. The product was amplified with the primers Yl_URA3_F2/Yl_URA3_R2 for the addition of *Not*I sites upstream and downstream of the *loxP-URA3* cassette. The PCR product was digested with *Not*I and ligated with the plasmid backbone, resulting in plasmid pFA6aURA3-09.

The deletion cassette for glycogen synthase was constructed according to the procedure described by Fickers *et al.* (2003). In brief, 1 kb of the promoter (*YlGSY1*^P^) and terminator (*YlGSY1*^T^) regions of the *YALI0F18502g* ORF were amplified with primer pairs Yl_GSYP_F/Yl_GSYP_R and Yl_GSYT_F/Yl_GSYT_R, respectively, and sequentially cloned into the plasmid pFA6aURA3-09, resulting in pGMKGSY_12. The correct assembly of the episomal *YlGSY1^P^-loxP-URA3-loxP- YlGSY1^T^* cassette was confirmed by sequencing. This cassette was excised from the plasmid by *Not*I digestion, gel-purified and used for transformation of JMY322. The integration of the cassette at the correct locus and loss of the *YALI0F18502g* ORF was confirmed by control primer PCR and sequencing.

The same strategy was used to delete *YALI0F18502g* in the TAG synthesis-deficient strain but we failed to obtain deletion strains in several attempts. Therefore, we used a CRISPR/Cas9-based approach as described by Schwartz *et al.* (2016) with the sequence from 27–46 bp in the putative *GSY* reading frame as guide sequence. The insertion of the guide sequence into the plasmid pCRISPRyl was performed according to Schwartz *et al.* (2016). In brief, an equimolar mixture of the primers CRSPyl_gsy_f and CRSPyl_gsy_r was heated up to 95°C and allowed to cool down to room temperature at a rate of 1°C/min, to obtain a double-stranded DNA bearing the guide sequence and flanking sequences for Gibson assembly with the linearized plasmid pCRISPRyl. A total of 1.5 μg of the resulting plasmid, pCRISPRyl/GSY, was used to transform the TAG-deficient mutant strain according to Le Dall, Nicaud and Gaillardin (1994). After transformation, the cells were inoculated into selection medium (-leu) and incubated for 48 h. Aliquots were plated onto YPD plates to obtain single colonies, which were stained with Lugol's solution (1% KI, 0.5% I_2_) to identify glycogen-deficient mutants. *YALI0F18502g* was amplified from genomic DNA of these colonies with the primers Yl_GSY_F/Yl_GSY_R and sequenced with the primer Yl_GSYT_Ctr_R to confirm a frame shift in the target region of the gene*.*

### Construction of the obese mutants

The previously described plasmids (Lazar *et al.*^2014^), JME1364 (*tgl4*Δ), JME1822 (pTEF-*DGA2*), JME1128 (pTEF-*GPD1*) and URA3ex, were *Not*I digested for excision of the desired DNA fragments. In addition, for the construction of the obese strain deleted in Yl*GSY1*, the plasmid pGMKGSY_12 was used as described above. The linearized DNA fragments were used to transform *Y. lipolytica* and transformants were selected on YNBUra, YNBLeu or YNB, depending on their genotype. Positive transformants were identified by PCR and confirmed by sequencing. The recycling of the selection markers was carried out by using the *LoxP*-Cre system as described by Fickers *et al.* (2003).

### Analytical methods

Biomass determination: cells densities were measured with a Casy^®^ TTC cell counter equipped with a 60 μm capillary (Roche Diagnostics GmbH, Roche Applied Science, Penzberg, Germany). The cell dry weights were determined by filtration through 0.45 μm nitrocellulose filters (Sartorius Stedim, Goettingen, Germany) and subsequent drying at 97°C overnight.

Analysis of metabolites: media analyses for determination of extracellular metabolites (glucose, glycerol, mannitol, citrate, succinate, acetic acid and ethanol) were performed with an HP 1100 series HPLC system equipped with an Aminex HPX-87H column (Biorad, Richmond, CA, USA), a UV detector (Agilent Technologies Österreich GmbH, Vienna, Austria) and a Knauer Differential refractometer as explained by Hanscho *et al.* (2012). During cultivations, 1 mL of culture was harvested, centrifuged at 16 000 g at 4°C for 1 min and the supernatant was directly injected into HPLC or stored at –80°C until analysis.

Glycogen and trehalose analyses and lipid extractions were performed according to Hanscho *et al.* (2012). Fatty acid methyl esters for GC-MS or GC-FID were prepared from 200 μL of lipid extract according to Kavšček *et al.* (2015). Heptadecanoid (C17:0) acid was used as an internal standard.

All averaged data and their standard deviations are derived from a minimum of three biologically independent experiments. If it was necessary to increase the number of independent experiments to confirm significance of differences, this is mentioned in the Results section.

### Flux balance analysis

The *Y. lipolytica i*MK735 genome scale metabolic model (Kavšček *et al.*^2015^) was used to predict changes in fluxes for a strain deleted for glycogen synthesis. An optimization was done in the MATLAB environment using the COBRA toolbox (Schellenberger *et al.*^2011^). The glycogen production rate and glycerol uptake rates were calculated from the experimental data and used as constraints in optimization for lipid production in the wild-type model. For the calculation of the effects of *gsy* deletion, the glycogen production rate was set to zero and the same glycerol uptake rate as in the wild-type model was used. A dynamic FBA calculation with 3 g L^−1^ cell dry weight was made to calculate the final TAG content.

## RESULTS AND DISCUSSION

### Glycogen contributes up to 16% to the biomass of *Yarrowia lipolytica*

*Yarrowia lipolytica* is well known and intensively investigated for its ability to accumulate high amounts of storage lipid under conditions where the carbon source is available in excess but another nutrient, typically the nitrogen source, is limiting. However, the behavior of this yeast regarding accumulation of the second important storage metabolite, glycogen, has not been investigated in detail. To assess the effects of different nutrient limitations on glycogen accumulation in *Y. lipolytica*, we performed shake flask cultivations under carbon as well as under nitrogen-limited conditions. For the carbon-limited cultivations, 5 g L^−1^ glucose or glycerol were used to avoid physiological stress due to a decrease of the pH when cells are growing to high densities, as it is the case in media with 20 g L ^−1^ carbon source. For nitrogen limitation, 20 g L^−1^ of the carbon source were used, but the ammonium sulfate concentration was reduced from 5 to 0.4 g L^−1^, resulting in depletion of the nitrogen source when <50% of the carbon source are consumed (Kavšček *et al.*^2015^).

**Figure 1.**
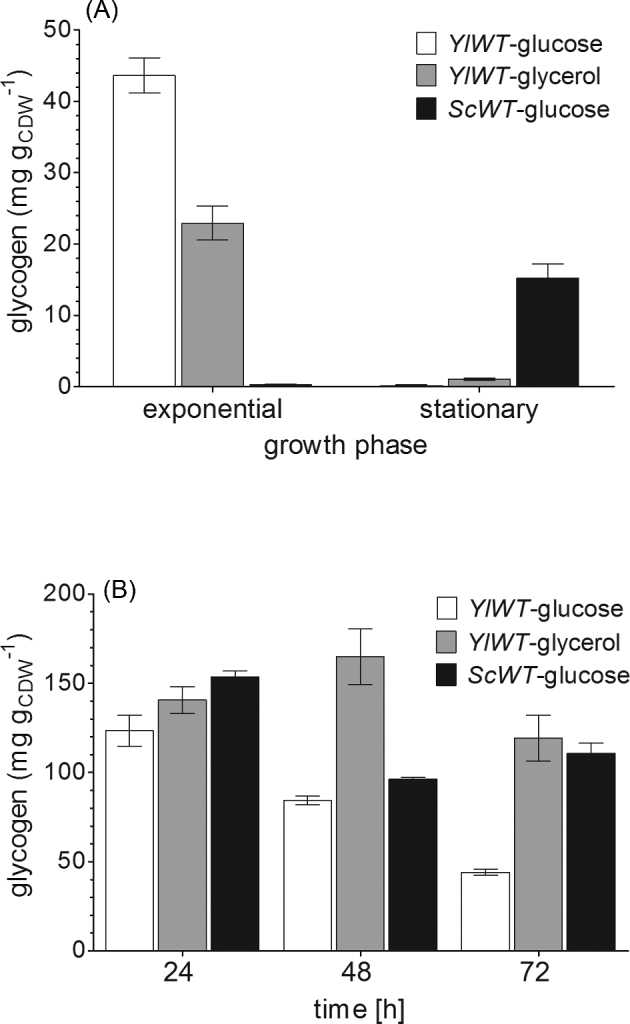
Glycogen accumulation in wild-type strains of *Y. lipolytica* and *S. cerevisiae.* (**A)** Glycogen content in the biomass of *Y. lipolytica* and *S. cerevisiae* during cultivation in carbon-limited media. *Yarrowia lipolytica (YlWT)* accumulated only moderate amounts of glycogen in the exponential phase of growth. These storage pools were depleted in the early stationary phase. In contrast, the glycogen levels in *S. cerevisiae* were low during exponential growth, but increased in the stationary phase. (**B)** Glycogen content of *Y. lipolytica* and *S. cerevisiae* during cultivation in nitrogen-limited media. Under lipogenic conditions, both yeasts accumulated high amounts of glycogen.

The wild-type strain accumulated 44 ± 2 and 23 ± 2 mg glycogen per g_CDW_ (CDW, cell dry weight) during the exponential growth phase in glucose and glycerol containing media, respectively. These stores were depleted during stationary phase. Interestingly, the opposite behavior was observed in *Saccharomyces cerevisiae*, where glycogen accumulates only in the stationary phase (Fig. 1A). Under nitrogen-limited conditions, a significant increase in glycogen content was observed for both yeast species, with a maximum of 165 mg g_CDW_^−1^ (Fig. 1B) in *Y. lipolytica*. Hence, in addition to its oleaginous phenotype, *Y. lipolytica* is able to accumulate high amounts of carbohydrate stores. Remarkably, the dynamics of glycogen storage are similar to those of lipid storage, with low levels during exponential growth and a strong increase under nitrogen-limited, i.e. lipogenic, conditions.

*Yarrowia lipolytica* is mainly investigated for its oleaginous phenotype. A multitude of different genetic engineering and fermentation strategies have been applied to increase the lipid content of this yeast (Blazeck *et al.*^2014^; Dulermo *et al.*^2015^; Kavšček *et al.*^2015^; Rakicka *et al.*^2015^; Friedlander *et al.*^2016^). Since *Y. lipolytica* accumulates more than 16% glycogen in its biomass under the conditions that are typically used for lipid accumulation (Fig. 1B), we wanted to know whether suppression of glycogen storage might result in an increase of the TAG content, due to a redirection of the carbon flux from glycogen to lipid synthesis. We used flux balance analysis (FBA) with a recently published network reconstruction of *Y. lipolytica* (Kavšček *et al.*^2015^) to investigate the theoretically possible effect of the elimination of glycogen storage on a computational level. Such a modeling approach allows for the optimization of a user-defined function. In our case, we calculated the optimal solution for the maximization of lipid synthesis. In a ‘wild-type model’, growing on glycerol as carbon source, we obtained a TAG synthesis rate of 15.9 μmol g^−1^ h^−1^ (corresponding to ca. 0.3 g TAG per g_CDW_ in 24 h). If glycogen synthesis was eliminated by setting the glycogen content in the biomass to zero, the flux into TAG increased to 17.0 μmol g^−1^ h^−1^, thus predicting an improvement of the synthesis rate by 7.1%.

In practice, FBA is often not able to predict the exact quantitative responses to the change of model parameters because regulatory mechanisms, such changes on the transcriptional or post-translational level, are not implemented in the network reconstruction. Nevertheless, such predictions can serve as guidelines for the optimization of strains by genetic modifications. Therefore, we concluded that the deletion of glycogen synthesis might be a promising strategy to increase NL synthesis in *Y. lipolytica*.

### Glycogen synthase activity is encoded by a single non-essential gene in *Yarrowia lipolytica*

**Figure 2.**
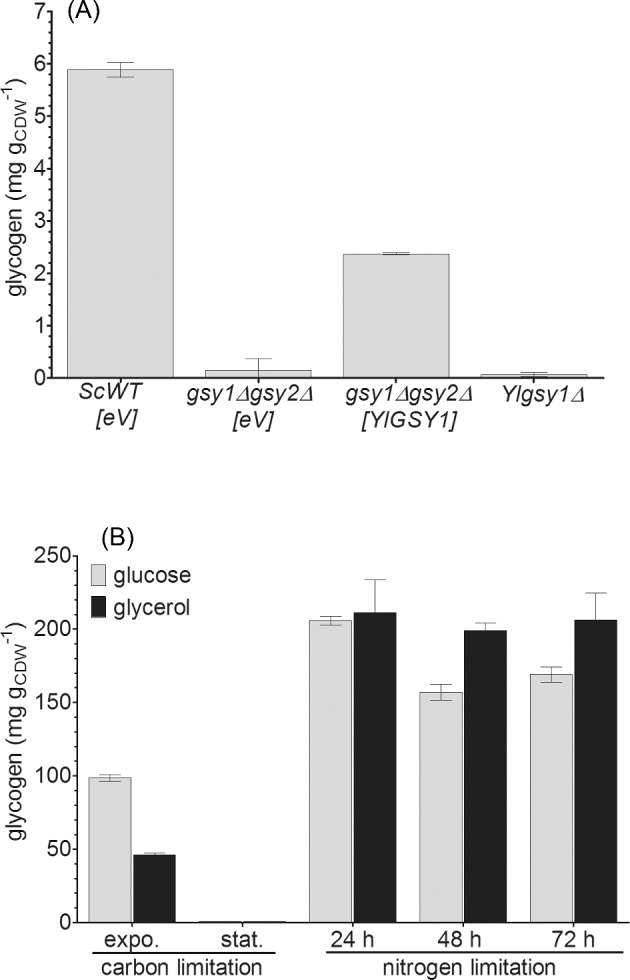
*YALI0F18502g* encodes the only glycogen synthase in *Y. lipolytica.* (**A)** The episomal expression of *YALI0F18502g* in a *S. cerevisiae* mutant deleted for *GSY1* and *GSY2* resulted in the reconstitution of glycogen storage, confirming that *YALI0F18502g* is indeed a glycogen synthase. The deletion of this reading frame in *Y. lipolytica* resulted in a complete loss of glycogen synthesis under both carbon and nitrogen limitation. Data are shown for cells grown in carbon-limited media with glucose as carbon source. [*eV*] empty vector, [*YlGSY1*] episomal expression of *YALI0F18502g, Ylgsy1Δ Y. lipolytica* bearing a deletion of the glycogen synthase. (**B**) In a *Y. lipolytica* strain overexpressing the glycogen synthase gene, glycogen storage is significantly increased during cultivation in nitrogen-limited media. The degradation of glycogen, as it was observed in the wild-type (Fig. 1B), was strongly delayed or compensated for by *de novo* synthesis.

The genome of *Y. lipolytica* bears one CDS that was annotated as glycogen synthase, due to its similarities with Gsy1 and Gsy2 of *S. cerevisiae* (Dujon *et al.*^2004^). In a pairwise sequence alignment (Smith and Waterman 1981), the encoded protein, YALI0F18502p, is 67% identical with the two glycogen synthases of baker's yeast. Therefore, we will use the name YlGsy1p for this protein in the following sections. To confirm its glycogen synthase activity by complementation of an *S. cerevisiae* glycogen synthase-deficient strain, we amplified *YlGSY1* from *Y. lipolytica* and cloned it into an *S. cerevisiae* expression vector under the control of a strong constitutive promoter. We deleted *GSY1* and *GSY2* in an *S. cerevisiae* wild-type strain and transformed this double mutant, which does not accumulate any glycogen, with the vector bearing *YlGSY1*. As shown in Fig. 2A, heterologous expression of this gene resulted in reconstitution of glycogen storage in the *gsy1Δ gsy2Δ* double mutant.

Furthermore, we deleted *YlGSY1* in JMY322. Our analysis showed that the resulting *Ylgsy1Δ* mutant strain does not accumulate detectable amounts of glycogen (Fig. 2A), neither in carbon-limited media during exponential growth nor in nitrogen-limited media, where glycogen accounts for up to 16% of the biomass. Taken together, these results confirm that *YlGSY1* is the only gene coding for a glycogen synthase in *Y. lipolytica*.

Finally, we overexpressed *YlGSY1* and analyzed the resulting mutant for its properties regarding glycogen accumulation during growth in both carbon- and nitrogen-limited media (Fig. 2B). During exponential growth in carbon-limited media, the glycogen content was two times higher than in the wild-type but the overexpression of *YlGSY1* had no influence on the complete degradation of glycogen during stationary phase, as it was also observed for the wild-type (shown in Fig. 1A). Under nitrogen limitation, however, the decrease in glycogen content, which was more pronounced in glucose than in glycerol media, was delayed in the *YlGSY1* overexpressing strain, resulting in an up to 4-fold glycogen content compared to the wild-type.

### Deletion of glycogen synthesis in *Yarrowia lipolytica* results in increased TAG accumulation

**Figure 3.**
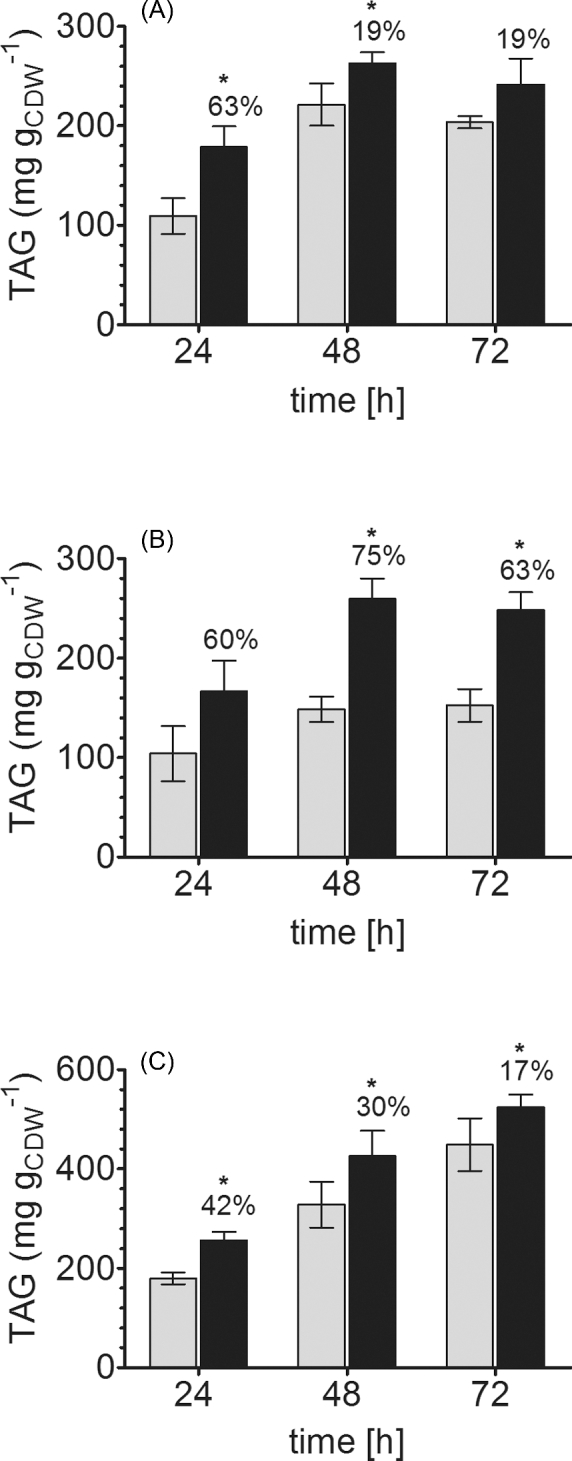
Deletion of glycogen synthase results in improved TAG synthesis. (**A** and **B)** Lipid content of *Y. lipolytica* wild-type and *gsy1Δ* mutant grown in nitrogen-limited glucose (A) and glycerol (B) media. Under all conditions tested, the deletion of *GSY1* resulted in an improvement of lipid accumulation, with the highest increase (+75%) after cultivation in glycerol for 48 h. (**C**) The deletion of *YlGSY1* in the obese background resulted in the same trend, although with lower differences than in the wild type, with the maximal difference (+42%) after 24 h. The highest lipid content of 524 mg TAG per g_CDW_ was obtained after 72 h. * P ≤ 0.05 in a two-tailed *t*-test. Gray bars: wild-type for panels A, B and ‘obese’ for panel C, black bars: respective *gsy1* deletion strains.

To experimentally test the predictions obtained with FBA, we cultivated the *Y. lipolytica* wild type and the *Ylgsy1*Δ mutant strain under nitrogen-limited conditions, to induce lipid accumulation. For both glucose and glycerol as carbon sources, we observed a significant improvement in lipid accumulation in the mutant, as compared to the wild-type strain. After 24 h of cultivation, the reference strain accumulated 110 ± 18 and 104 ± 28 mg TAG per g_CDW_ on glucose and glycerol as carbon source, respectively. Under the same conditions, we obtained TAG values of 179 ± 21 and 167 ± 31 mg per g_CDW_ in the *Ylgsy1Δ* mutant, corresponding to a more than 60% increase in TAG synthesis for both carbon sources (Fig. 3). Similar differences were observed at later time points, although they were more pronounced in glycerol than in glucose. The highest TAG content of 26% was obtained with the *Ylgsy1Δ* mutant cultivated in glycerol medium for 48 h.

The effect of the *Ylgsy1* deletion on TAG accumulation was not observed when the cells were grown in carbon-limited medium, where the oleaginous phenotype is not induced. In fact, the TAG content in stationary phase was even reduced in the *Ylgsy1Δ* mutant, as compared to the wild-type (see Table S2, Supporting Information). Furthermore, lipid analysis of *S. cerevisiae* strains grown in nitrogen-limited medium showed that the deletion of *GSY1* and *GSY2* resulted in less pronounced or even no changes in lipid content of this species (see Table S3, Supporting Information). We speculate that this difference can be attributed to the fact that in the Crabtree-positive *S. cerevisiae*, most of the catabolized glucose is diverted to ethanol for regeneration of NAD^+^, whereas in the strictly respiratory *Y. lipolytica* glucose is converted to biomass—in this case TAG—more efficiently.

The increase in the TAG content of the *Y. lipolytica gsy1Δ* mutant was mainly due to an increase of desaturated C18 FAs. Indeed, the level of C16:0 remained almost unchanged, whereas C18:1 levels increased up to 2-fold (see Table S4, Supporting Information). Hence, the deletion of glycogen synthesis seems to cause an increase of FA *de novo* synthesis and of FA desaturation activity.

Interestingly, the improvement in TAG accumulation in the deletion mutant was higher than it would have been expected from a simple redirection of the carbon flux from glycogen to FA synthesis. For example, the 119 mg glycogen that are stored in the wild-type after 72 h of cultivation in glycerol (Table 2) would allow for the synthesis of 36.5 mg TAG if this carbon flux were redirected to TAG with the maximum theoretical yield (Ratledge 2014). However, the glycogen-deficient mutant produces ca. 100 mg more TAG than the wild-type at the same time point. Although this difference is lower in glucose-grown cells than in a glycerol medium, it was observed under all conditions. In *S. cerevisiae*, the glucose dimer trehalose plays an important role in storage of carbohydrates. To investigate whether trehalose might contribute to the changes in TAG content of the glycogen-deficient mutant of *Y. lipolytica*, we quantified this metabolite in the wild-type and in the mutant under the same conditions as they were used for TAG analysis. These experiments showed that the trehalose content always remains below 1 mg g_CDW_^−1^ (Table 2). Although it is further reduced to almost zero in the *Ylgsy1Δ* mutant, this reduction cannot explain the observed increase of the TAG content in the mutant, indicating that the deletion of *YlGSY1* has an additional—unknown—effect on lipid synthesis, beyond the mere redirection of the carbon flux from glycogen to TAG.

**Table 2.** Physiological data for wild-type and deletion mutant in nitrogen-limited media.

	*YlWT*			*Ylgsy1Δ*		
	24 h	48 h	72 h	24 h	48 h	72 h
**I. Glucose**						
Biomass (g L^−1^)						
CDW	2.4 ± 0.2	3.4 ± 0.2	3.5 ± 0.1	2.0 ± 0.1	3.2 ± 0.2	3.4 ± 0.1
Extracellular metabolites (g L^−1^)						
Glucose	14.7 ± 0.6	9.4 ± 0.4	6.2 ± 0.3	15.4 ± 0.3	10.1 ± 0.1	6.9 ± 0.1
Citrate	n.d.	0.4 ± 0.1	1.3 ± 0.2	n.d.	0.1 ± 0.0	1.1 ± 0.1
Succinate	0.2 ± 0.0	0.2 ± 0.0	n.d.	0.2 ± 0.0	0.2 ± 0.0	n.d.
Mannitol	0.5 ± 0.2	1.6 ± 0.3	2.2 ± 0.3	0.4 ± 0.1	1.3 ± 0.1	1.7 ± 0.1
Intracellular metabolites (mg g_CDW_^−1^)						
Trehalose	0.33 ± 0.14	0.09 ± 0.05	0.10 ± 0.02	0.06 ± 0.04	n.d.	0.02 ± 0.00
Glycogen	123 ± 9	84 ± 2	44 ± 2	n.d.	n.d.	n.d.
TAG	110 ± 18	221 ± 21	204 ± 6	179 ± 21*	263 ± 11*	242 ± 26
**II. Glycerol**						
Biomass (g L^−1^)						
CDW	2.6 ± 0.1	3.8 ± 0.0	4.0 ± 0.1	2.2 ± 0.1	3.3 ± 0.1	3.6 ± 0.1
Extracellular metabolites (g L^−1^)						
Glycerol	14.7 ± 0.8	7.7 ± 0.4	2.5 ± 0.3	14.9 ± 0.7	6.2 ± 0.4	n.d.
Citrate	n.d.	0.9 ± 0.1	2.1 ± 0.4	n.d.	1.3 ± 0.1	3.2 ± 0.7
Succinate	0.1 ± 0.1	0.1 ± 0.1	0.1 ± 0.0	0.1 ± 0.1	0.1 ± 0.1	n.d.
Mannitol	0.4 ± 0.0	2.9 ± 0.5	5.1 ± 0.9	0.7 ± 0.1	3.7 ± 0.5	6.2 ± 0.5
Intracellular metabolites (mg g_CDW_^−1^)						
Trehalose	0.60 ± 0.11	0.29 ± 0.02	0.18 ± 0.03	0.13 ± 0.06	0.03 ± 0.00	0.01 ± 0.01
Glycogen	141 ± 8	165 ± 16	119 ± 13	n.d.	n.d.	n.d.
TAG	104 ± 28	149 ± 13	152 ± 16	167 ± 31	260 ± 20*	248 ± 18*

n.d., not detected

* *P* ≤ 0.05 in unpaired two-tailed *t*-tests

Based on the assumption that yeast is able to compensate for changes in one storage pathway by adapting the flux through the remaining one, we analyzed the lipid content of YlTEFGSY1, the strain overexpressing *YlGSY1*. Contrary to our expectations, this strain, which accumulates high amounts of glycogen, does not store less NL. In fact, the TAG content of the mutant is the same as in the wild-type during cultivation in N-lim media and even higher than in wild-type in both exponential and stationary phase of growth in C-lim media (see Table 3 and Table S2). Likewise, the deletion of NL synthesis in *Y. lipolytica* did not result in an increase of glycogen storage, as it would have been expected assuming a compensation mechanism (Table S5, Supporting Information). Indeed, the glycogen content was reduced in the NL-deficient quadruple mutant (JMY1877, Table 1). On the other hand, the deletion of NL synthesis in baker's yeast resulted in the expected effect. This strain accumulated several-fold more glycogen and trehalose than the wild-type parent strain, although such a significant difference was only observed during exponential growth and not under N-lim conditions.

**Table 3.** Glycogen and TAG accumulation (mg g_CDW_^−1^) in the mutant overexpressing *YlGSY*.

Metabolite		Glycogen		TAG	
Carbon source		Glucose	Glycerol	Glucose	Glycerol
C-lim	Exponential	99 ± 2	46 ± 1	61 ± 5	68 ± 4
	stationary	1 ± 0	1 ± 0	51 ± 7	51 ± 4
N-lim	24 h	206 ± 3	211 ± 22	135 ± 5	120 ± 10
	48 h	157 ± 5	199 ± 5	226 ± 3	135 ± 30
	72 h	169 ± 5	206 ± 18	203 ± 11	155 ± 7

Hence, alterations in one of the storage pathways result in changes in the second one in almost all cases and it seems clear that the two processes are connected to each other. However, a simple model assuming a compensatory strategy of the cell is not sufficient to explain our observations. Furthermore, the different responses of *S. cerevisiae* and *Y. lipolytica* to modifications in the pathways for TAG and glycogen synthesis indicate that storage metabolism is regulated by different mechanisms in these two yeasts.

### Elimination of glycogen storage in an obese mutant results in further improvement of lipid accumulation

From a biotechnological perspective, the reduction or, if possible, deletion of competing pathways is a standard approach to improve product yields. We have shown that glycogen synthesis is indeed a competing process with respect to TAG synthesis, despite the distance of the two pathways in the cellular metabolic network. Therefore, we investigated the impact of the deletion of *YlGSY1* in a strain background that is already optimized for TAG production with known effectors of this pathway. This strain was obtained by deletion of the gene coding for TAG lipase, *TGL4*, and overexpression of *DGA2* and *GPD1*, the genes encoding diacylglycerol transferase and glycerol-3-phosphate dehydrogenase, respectively (Dulermo and Nicaud 2011; Beopoulos *et al.*^2012^; Lazar *et al.*^2014^). When cultivated in nitrogen-limited media, this strain, named ‘*obese*’, stores up to 449 ± 53 mg TAG and up to 181 ± 8 mg glycogen per g_CDW_ (Table 4). We deleted *YlGSY1* in this strain background and confirmed the loss of its ability to store glycogen. Cultivation in N-lim media and subsequent lipid analysis showed that this deletion has similar effects in the *obese* background as in the wild-type. After 72 h, the biomass of *obese gsy1Δ* contained 524 ± 25 mg TAG, corresponding to a 17% improvement in comparison to the already obese parent strain (Fig. 3C and Table 4). As opposed to the wild-type background, the deletion of *YlGSY1* in the *obese* background did not result in an increase of unsaturated FA only, but in an equal improvement of all species, keeping the degree of saturation approximately constant (see Table S4).

**Table 4.** Physiological data for *obese* and *obese gsy1Δ* in nitrogen-limited glycerol medium.

	*obese*			*obese gsy1Δ*		
	24 h	48 h	72 h	24 h	48 h	72 h
Biomass (g L^−1^)						
CDW*	2.6 ± 0.3	4.5 ± 0.2	5.5 ± 0.4	2.0 ± 0.2	3.6 ± 0.2	5.0 ± 0.4
Extracellular metabolites (g L^−1^)						
Glycerol	14.4 ± 0.3	8.2 ± 0.1	3.0 ± 0.2	15.1 ± 0.3	8.2 ± 0.1	2.1 ± 0.1
Citrate	n.d.	n.d.	n.d.	n.d.	n.d.	0.1 ± 0.0
Succinate	0.3 ± 0.0	0.4 ± 0.0	1.7 ± 2.2	0.3 ± 0.0	0.5 ± 0.0	0.6 ± 0.0
Mannitol	0.2 ± 0.0	1.1 ± 0.1	1.9 ± 0.1	0.4 ± 0.0	1.6 ± 0.0	2.5 ± 0.0
Intracellular metabolites (mg g_CDW_^−1^)						
Trehlaose	1.64 ± 0.25	0.93 ± 0.01	0.62 ± 0.06	0.09 ± 0.01	0.09 ± 0.07	n.d.
Glycogen*	181 ± 8	124 ± 24	85 ± 19	n.d.	n.d.	n.d.
TAG*	180 ± 12	328 ± 46	449 ± 53	256 ± 17**	426 ± 51**	524 ± 25**

n.d., not detected

* Values from five independent biological replicates.

** *P* ≤ 0.05 in unpaired two-tailed *t*-tests.

### The chronological lifespan of *Yarrowia lipolytica* is affected by a loss of storage metabolism

The mutant strains of *Y. lipolytica* overexpressing glycogen synthase or deleted for this gene did not show any measurable differences to the wild-type with regard to growth rate, biomass yield or cell morphology. Furthermore, like in the wild-type, we did not observe any detectable amounts of metabolites excreted into the medium during growth in carbon-limited media (data not shown).

**Figure 4.**
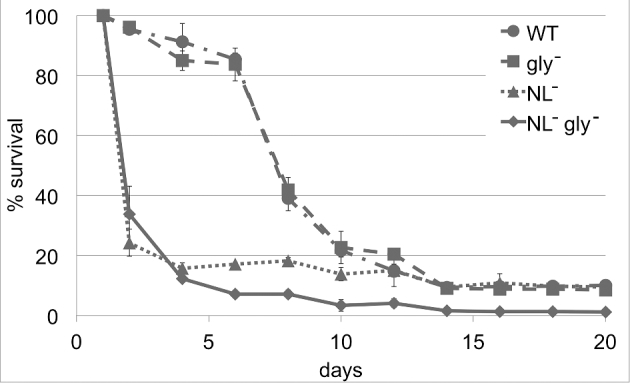
Chronological lifespan is affected by the loss of storage metabolism. The *Y. lipolytica* wild-type strain (WT) and its derivatives deleted for glycogen (gly^−^) or neutral lipid (NL^−^) synthesis or both (NL^−^ gly^−^) were cultivated until depletion of glucose (C-lim minimal medium), and the survival rate was determined by plating diluted samples. The viability of the NL-deficient strain was strongly reduced in comparison to its parent and the additional deletion of *YlGSY1* in this mutant, resulting in a strain without the ability to store carbon, caused a further reduction of the survival rate.

Because storage metabolism is often assumed to play a role in survival during stationary phase, we also assessed the chronological lifespan of the deletion mutant. These experiments indicated that the inability to store glycogen does not affect viability of *Y. lipolytica* over 20 days of starvation (Fig. 4). A similar behavior was demonstrated by Osório *et al.* (2005) for *S. cerevisiae*, and we confirmed these results for our strain background and growth conditions (Fig. S1, Supporting Information). Viability has also been shown for NL-deficient mutants, deleted for both TAG and steryl ester (SE) synthesis (Sandager *et al.*^2002^; Beopoulos *et al.*^2012^), but the effect of a complete deletion of storage metabolism has not yet been investigated. Therefore, we deleted glycogen synthesis in the NL-deficient mutant backgrounds of both *Y. lipolytica* and *S. cerevisiae*. We obtained viable strains for both species and confirmed that both glycogen and NL levels were below the limit of detection. Subsequent chronological life span experiments showed that the deletion of storage lipid synthesis in *Y. lipolytica* has a strong negative effect on viability during stationary phase, with already more than 80% dead cells after only 4 days of starvation. For the mutant deleted for both NL and glycogen synthesis, we found a similar behavior during this first period. In the following days, the viability of the NL-deficient strain remained constant, whereas the survival rate of the completely storage-deficient strain further declined, with only ca. 1% surviving cells after 20 days (Fig. 4). In contrast, no differences between wild-type and mutants were observed for *S. cerevisiae* (Fig. S1).

As for the *Y. lipolytica* strain that was deleted only for glycogen synthesis, the pentuple mutant (YlPM, Table 1) did not compensate for the loss of both NL and glycogen synthesis by accumulating trehalose, neither during growth in C-lim nor in N-lim media (Table S5). Hence, this strain is completely devoid of any form of carbon storage, confirming that storage metabolism is not essential. It has to be noted, however, that already the deletion of TAG and SE synthesis causes a severe growth defect during exponential growth in C-lim media (maximum specific growth rate μ = 0.27 h^−1^ for wild-type and 0.08 h^−1^ for the NL-deficient mutant), a phentoype that was also observed for the NL-deficient *S. cerevisiae* mutant. This phenotype was not further aggravated by the additional deletion of glycogen synthesis, suggesting that the storage of NLs plays a more important role than glycogen. This finding might reflect the fact that the lipids stored in the ‘inert’ lipid droplet play a crucial role in homeostasis and turnover of membrane lipids in yeasts, rather than just serving as energy reserve.

## CONCLUSION

*Yarrowia lipolytica* accumulates large amounts of glycogen during cultivation under conditions that are typically used for lipid accumulation, and the same might be assumed for other oleaginous yeasts. Hence, although the two pathways for TAG and glycogen synthesis are not closely connected to each other, glycogen synthesis is a competing reaction during TAG accumulation and should be suppressed to maximize the yield, even in strain backgrounds that have already been genetically engineered for high lipid yields. In addition, it might be speculated that not only FA synthesis but also other production pathways, like the synthesis of terpenoids or polyketides, would benefit from elimination of glycogen storage. Importantly, the deletion of *GSY1* causes not only a redirection of the carbon flux that is normally bound for glycogen synthesis towards FA production. Rather, it induces an additional increase in TAG synthesis beyond what would be expected from the mere rearrangement of carbon storage pools, resulting in higher rates and yields. Moreover, the results of this study suggest that the loss of glycogen storage has no significant impact on the growth rate and viability of yeast strains. Indeed, for both the respiratory and oleaginous *Y. lipolytica* as well as for the fermentative *S. cerevisiae*, the storage of lipids seems to play a more important role than of glycogen.

## Supplementary Material

Supplemental material Supplementary data are available at *FEMSYR* online.
