# Supplementary material for: Sugar versus fat: elimination of glycogen storage improves lipid accumulation in Yarrowia lipolytica
Source: FEMS Yeast Res. 2017 Apr 17;17(3):fox020. doi: 10.1093/femsyr/fox020 (PMC5812513; doi:10.1093/femsyr/fox020)
Supplement: Supplemental material — Supplementary data are available at FEMSYR online. [file fox020_supp.docx]

**Supplemental Material**

**Table S1.** Primers used in this study

| Primer | Sequence 5' to 3' |
| --- | --- |
| Yl_URA3_F1 | CAGCTGAAGCTTATAACTTCGTATAGCATACATTATACGAAGTTATCGCCCAGAGAGCCATTGAC |
| Yl_URA3_R1 | CGACATCGATATAACTTCGTATAATGTATGCTATACGAAGTTATGAGAAACACAACAACATGCCC |
| Yl_URA3_F2 | GCCATGCGGCCGCGGATCCGCATGCACTAGTCAGCTGAAGCTTATAACTTCGTAT |
| Yl_URA3_R2 | GGCATGCGGCCGCTCTAGAAGATCTGAATTCGTCGACATCGATATAACTTCGT |
| Yl_GSYP_F | GCCATGCATGCCGAGACGGGCCTGATCCGAG |
| Yl_GSYP_R | GACGTAAGCTTGTTGGAATCGGTTGTTGCGAGAG |
| Yl_GSYT_F | GCCATATCGATGCAGAAGTTGAGTGTTTGAATGTAC |
| Yl_GSYT_R | GACGTTCTAGACCGTACACACTGTCGCATCTC |
| Yl_GSY_F | GCCATGAATTCATGACGAGGGACACACGAAACC |
| Yl_GSY_R | GACGTTCTAGACTACTTTCTGCTACCGGCTAGAG |
| gsy1_del_f | GAAACCTGTGAAGAAAAAGAAAATAAACTCAGACGCAGCATCACAGCGAAGTGCGGCCGCCAGGGG |
| gsy1_del_r | ATTTGATGCTCAGCTAAAAGAGTAAGATATGTTAGCAGAAGTTAAGATGGTTCACCTTTAGACATGATGGG |
| gsy2_del_f | GTTTTTTTGATAACTGTGATTGAAGTTTTGACTACCTCAGAGAAAAATTTTGAGCGGCCGCCAGGGG |
| gsy2_del_r | CGTATGTCTTTATGGTAAGATTTTTTTAATACTGTTTATATCCTCATAGGATCACCTTTAGACATGATGGG |
| ura3_del_f | TCTTAACCCAACTGCACAGAACAAAAACCTGCAGGAAACGAAGATAAATCGCGGCCGCCAGGGG |
| ura3_del_r | GCTCTAATTTGTGAGTTTAGTATACATGCATTTACTTATAATACAGTTTTCACCTTTAGACATGATGGG |
| TEF-fwd | CCCATCCCCGGGACTAGTGCCGGCCCCACACACCATAGCTTCAAAATG |
| TEF-rev | ACGTAGGCATGCGGATCCGCGGCCGCCAGCTGGGTACCGTCGACGACGTCGAATTCTTTGTAATTAAAACTTAGATTAGATTGCTATGC |
| CRSPyl_gsy_f | GATTCCGGGTCGGCGCAGGTTGGCTGTTCGAGGTCGCCACCGGTTTTAGAGCTAGAAATAGC |
| CRSPyl_gsy_r | GCTATTTCTAGCTCTAAAACCGGTGGCGACCTCGAACAGCCAACCTGCGCCGACCCGGAATC |
| YlTEF1_GSYoe_F | GCATACATTATACGAAGTTATATAGAGACCGGGTTGGCGGCGCA |
| YlTEF1_GSYoe_R | CAAACACTCAACTTCTGCATCGATTTTGAATGATTCTTATACTCAGAAGGAAATGCTTAA |
| GSYoe_F | GTATAAGAATCATTCAAAATCGATATGACGAGGGACACACGAAACC |
| GSYoe_R | CAAACACTCAACTTCTGCATCGATCTAAGCGTAATCTGGAACATCGTATGGGTACTTTCTGCTACCGGCTAGAGTG |
| Yl_GSYT_Ctr_R | GAACATGTGTGCGTTTTCACTTTCG |

**Table S2**. TAG accumulation (mg g_CDW_^-1^) in wild type *(YlWT)* and single mutant *Ylgsy1Δ* in carbon-limited medium.

| Carbon source | Glucose | | Glycerol | |
| --- | --- | --- | --- | --- |
| Growth phase | exponential | stationary | exponential | stationary |
| *YlWT* | 43 ± 2 | 36 ± 7 | 34 ± 3 | 41 ± 5 |
| *Ylgsy1*Δ | 51 ± 1 | 26 ± 7 | 39 ± 5 | 38 ± 7 |

**Table S3.** TAG accumulation (mg g_CDW_^-1^) in *S. cerevisiae* wild type (*ScWT)* and double mutant (*gsy1Δgsy2Δ*) in nitrogen-limited glucose medium.

|  | 24 h | 48 h | 72 h |
| --- | --- | --- | --- |
| *ScWT* | 86 ± 6 | 92 ± 6 | 91 ± 8 |
| *gsy1Δgsy2Δ* | 86 ± 7 | 103 ± 10 | 110 ± 2 |

**Table S4.** Fatty acid species composition (mg g_CDW_^-1^) and desaturation index (DI) in nitrogen-limited media.

| I. glucose |  |  |  |  |  |  |  |  |
| --- | --- | --- | --- | --- | --- | --- | --- | --- |
| Strain | Time | C16:0 | C16:1 | C18:0 | C18:1 | C18:2 | DI | TOTAL |
| *YlWT* | 24 h | 16.2 ± 4.7 | 4.9 ± 0.9 | 8.0 ± 2.8 | 57.7 ± 14.5 | 22.7 ± 3.2 | 4.6 ± 0.9 | 109.5 ± 18.0 |
| *YlWT* | 48 h | 41.4 ± 7.0 | 10.9 ± 1.4 | 23.7 ± 1.7 | 107.8 ± 14.0 | 37.6 ± 3.1 | 3.0 ± 0.2 | 221.4 ± 21.3 |
| *YlWT* | 72 h | 37.6 ± 2.4 | 12.7 ± 1.5 | 24.3 ± 1.3 | 90.8 ± 0.9 | 38.2 ± 3.4 | 2.9 ± 0.1 | 203.6 ±   6.1 |
|  |  |  |  |  |  |  |  |  |
| *Ylgsy1Δ* | 24 h | 17.5 ± 3.3 | 6.4 ± 0.4 | 11.4 ± 2.4 | 103.2 ± 16.3 | 40.3 ± 1.7 | 6.7 ± 0.8 | 178.8 ± 20.5 |
| *Ylgsy1Δ* | 48 h | 39.5 ± 5.9 | 14.1 ± 1.3 | 24.7 ± 2.5 | 147.0 ± 12.3 | 37.8 ± 9.4 | 3.7 ± 0.6 | 263.1 ± 10.8 |
| *Ylgsy1Δ* | 72 h | 34.9 ± 5.1 | 16.9 ± 1.8 | 22.0 ± 2.2 | 123.1 ± 15.4 | 44.6 ± 4.4 | 4.0 ± 0.3 | 241.5 ± 26.1 |
|  |  |  |  |  |  |  |  |  |
| II. glycerol |  |  |  |  |  |  |  |  |
| Strain | Time | C16:0 | C16:1 | C18:0 | C18:1 | C18:2 | DI | TOTAL |
| *YlWT* | 24 h | 14.1 ± 4.4 | 4.9 ± 1.1 | 9.8 ± 3.4 | 45.6 ± 13.2 | 29.6 ± 6.1 | 4.7 ± 0.7 | 104.1 ± 27.6 |
| *YlWT* | 48 h | 25.7 ± 2.5 | 7.2 ± 0.2 | 19.3 ± 2.9 | 63.6 ± 5.6 | 32.9 ± 2.3 | 3.1 ± 0.2 | 148.6 ± 12.7 |
| *YlWT* | 72 h | 27.5 ± 2.1 | 7.8 ± 0.2 | 21.1 ± 3.4 | 61.0 ± 7.8 | 35.0 ± 3.5 | 2.9 ± 0.2 | 152.5 ± 16.4 |
|  |  |  |  |  |  |  |  |  |
| *Ylgsy1Δ* | 24 h | 14.7 ± 3.2 | 5.7 ± 0.6 | 15.4 ± 3.5 | 88.7 ± 18.8 | 42.1 ± 6.1 | 6.0 ± 0.4 | 166.6 ± 30.9 |
| *Ylgsy1Δ* | 48 h | 30.7 ± 4.0 | 11.0 ± 1.1 | 30.3 ± 3.1 | 140.1 ± 12.4 | 47.6 ± 3.0 | 4.1 ± 0.4 | 259.7 ± 20.4 |
| *Ylgsy1Δ* | 72 h | 28.3 ± 4.7 | 12.5 ± 2.3 | 29.4 ± 2.7 | 130.1 ± 9.1 | 48.0 ± 3.8 | 4.1 ± 0.2 | 248.3 ± 18.1 |
|  |  |  |  |  |  |  |  |  |
| *obese* | 24 h | 41.7 ±   3.1 | 14 ± 1.5 | 16.8 ± 1.2 | 85.8 ± 6.7 | 21.6 ± 0.8 | 2.4 ± 0.0 | 180.0 ± 11.7 |
| *obese* | 48 h | 88.7 ± 11.3 | 28.8 ± 3.7 | 27.3 ± 4.8 | 162.4 ± 24.6 | 21.1 ± 2.4 | 2.0 ± 0.1 | 328.3 ± 46.1 |
| *obese* | 72 h | 126.9 ± 11.6 | 40.9 ± 5.1 | 36.0 ± 6.0 | 222 ± 29.3 | 22.7 ± 3.2 | 1.9 ± 0.1 | 448.5 ± 52.8 |
|  |  |  |  |  |  |  |  |  |
| *obese gsy1Δ* | 24 h | 53.9 ±  4.3 | 19.0 ± 1.7 | 23.0 ± 2.2 | 127.8 ±   8.7 | 32.6 ± 2.4 | 2.8 ± 0.1 | 256.3 ± 17.4 |
| *obese gsy1Δ* | 48 h | 102.6 ±  9.7 | 38.7 ± 4.3 | 32.6 ± 5.5 | 221.1 ± 29.4 | 30.9 ± 2.7 | 2.4 ± 0.1 | 425.8 ± 50.9 |
| *obese gsy1Δ* | 72 h | 136.2 ± 11.5 | 48.4 ± 3.0 | 41.5 ± 7.0 | 268.7 ± 16.5 | 29.7 ± 2.0 | 2.1 ± 0.3 | 524.4 ± 25.4 |

DI=((18:2*2)+18:1+16:1)/(18:0+16:0)

**Table S5:** Glycogen and trehalose accumulation (mg g_CDW_^-1^) in yeast mutants deficient for neutral lipid synthesis (NL^-^) or for both NL and glycogen synthesis (NL^-^ gly^-^). Note that glycogen values for the wild type reference strains differ from Figure 1 in the main text because different parent strains were used for the construction of the NL-deficient quadruple strains, which were the starting strains for constructing the NL^-^ gly^-^ strains.

| ***I. Y. lipolytica*** | Strain | Glycogen | Trehalose |
| --- | --- | --- | --- |
| C-lim exponential | wild type | 66 ± 11 | 0.5 ± 0.2 |
|  | NL^-^ | 26 ±   4 | 0.2 ± 0.1 |
|  | NL^-^ gly^-^ | n.d. | n.d. |
| N-lim 24h | wild type | 89 ± 16 | 0.8 ± 0.1 |
|  | NL^-^ | 56 ± 10 | 0.9 ± 0.0 |
|  | NL^-^ gly^-^ | n.d. | 0.2 ± 0.0 |
|  |  |  |  |
| ***II. S. cerevisiae*** | Strain | Glycogen | Trehalose |
| C-lim exponential | wild type | 2 ±   0 | 1.5 ±   0.5 |
|  | NL^-^ | 14 ±   2 | 37.4 ± 21.7 |
|  | NL^-^ gly^-^ | n.d. | 40.7 ± 10.9 |
| N-lim 24h | wild type | 36 ±   5 | 42.5 ±   2.6 |
|  | NL^-^ | 42 ± 19 | 49.9 ±   8.0 |
|  | NL^-^ gly^-^ | n.d. | 47.7 ±   7.6 |

n.d. not detected

**Construction of the *S. cerevisiae* sextuple mutant deficient in both neutral lipid and glycogen synthesis**

*GSY1* and *GSY2* were deleted in the quadruple mutant deficient in neutral lipid (NL) synthesis, YJP1078 (Petschnigg et al., 2009), by using a marker-free CRISPR/Cas9 strategy.

The plasmid pCRISPR_CAS9_gRNA.CAN1.Y was assembled by Gibson assembly from fragments encoding i) Cas9 under control of the *TEF1* promoter, amplified from the plasmid p414-tef1-cas9-cycT-NatR (DiCarlo et al., 2013) , ii) structural gRNA targeting the *CAN1* ORF, amplified from the plasmid p426-SNR52p-gRNA.CAN1.Y-SUP4 (DiCarlo et al., 2013), iii) a *NAT*-*R* cassette, conferring nourseothricin resistance and iv) a backbone for low copy expression in yeast and ampicillin selection in *E. coli*. This plasmid was used to delete *CAN1* in a *S. cerevisiae* wild type strain, confirming functional expression of the CRISPR/Cas9 system from a single low copy vector.

Subsequently, *NAT-R*, the *SNR52* promoter and the guide sequence for *CAN1* were removed from the plasmid and a *Not*I restriction site was introduced upstream of the gRNA sequence, resulting in the plasmid pCR_empty. For the construction of the deletion plasmids, the fragment encoding *NAT-R* and the *SNR52* promoter was amplified from pCRISPR_CAS9_gRNA.CAN1.Y with primers bearing overhangs for the introduction of guide sequences for targeting Cas9 to *GSY1* (base pairs 97-116) and *GSY2* (bp 24-43), respectively. These amplicons were Gibson-assembled with the *Not*I digested pCR_empty backbone, resulting in pCRISPR_CAS9_gRNA.GSY1 and pCRISPR_CAS9_gRNA.GSY2. All cloning steps were confirmed by sequencing.

The quadruple mutant YJP1078 was first transformed with pCRISPR_CAS9_gRNA.GSY1 together with the double stranded oligo DNA 5'- CTCTGTCTTGAAGTCCAAGGCTCCAGTCACTGTGGCACATACAGAGACAACTATACGCTGCTTGGACCTTTGAACAAGGCGAC-3', to introduce a frame shift at bp 113 of the reading frame of *GSY1*. Correct mutation in nourseothricin-resistant colonies was confirmed by sequencing. The plasmid loss was induced by growth in YPD without nourseothricin and the resulting strain was transformed with the plasmid pCRISPR_CAS9_gRNA.GSY2 together with the double stranded oligo 5'-CCTCAGAGAAAAATTTTGAATGTCCCGTGACCTACAAAACAATTTTTATTCGAGACTGCGACTGAGGTTGCTAATAGGGTTGGTG-3' for deletion of *GSY2* by introducing a frame shift at bp 27. The confirmation of correct mutation and the plasmid loss were performed as described for *GSY1*. In addition, glycogen analysis, as described in the Materials and Methods section of the main text, was performed to confirm glycogen deficiency of the sextuple mutant.

This storage-deficient strain, its NL-deficient parent strain, a glycogen deficient strain and the wild type were analyzed for chronological life span. In contrast to the *Yarrowia lipolytica* mutants (see main text), no differences in survival could be found during the observed time span (Figure S1).

**
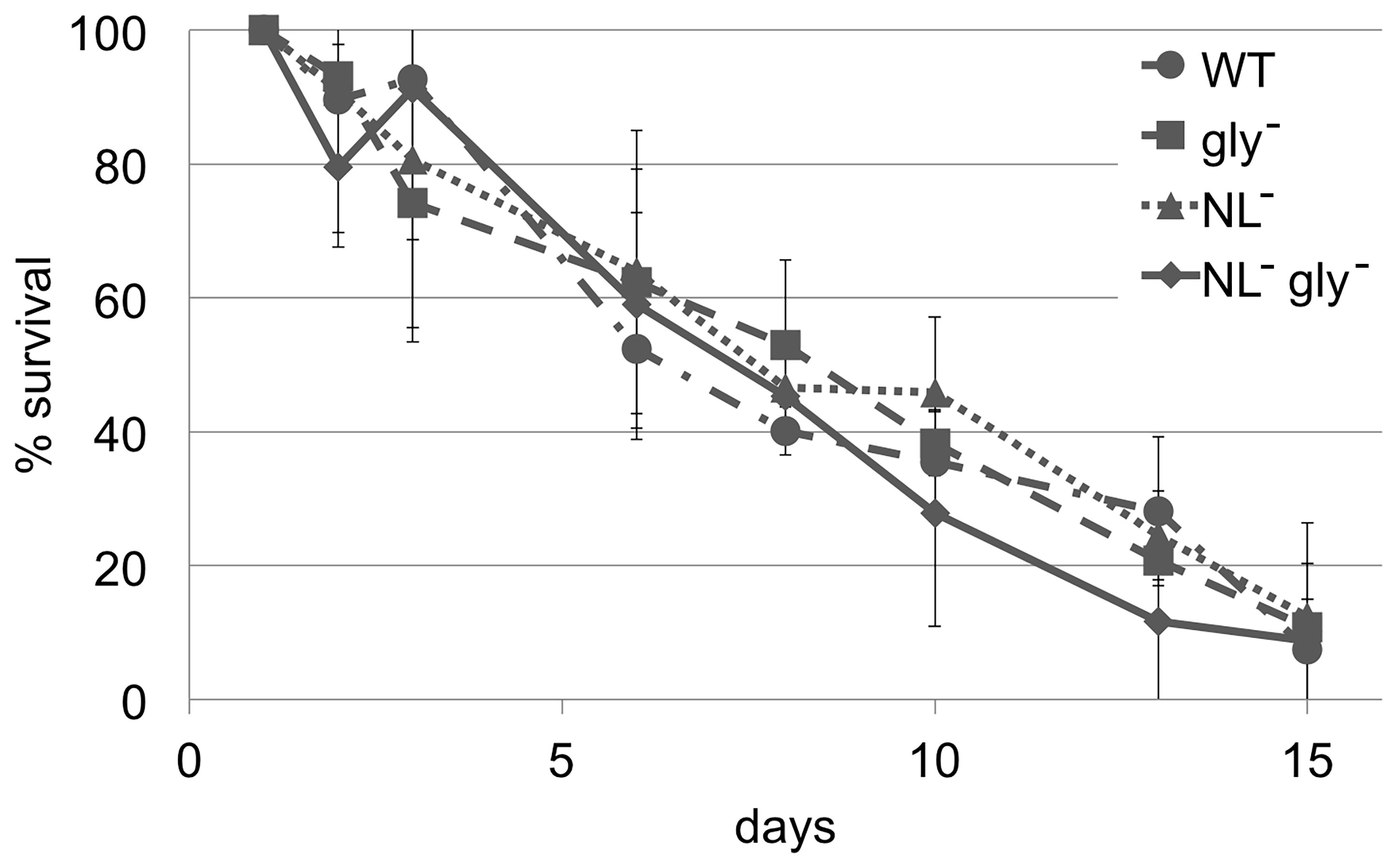
**

**Figure S1: Survival of *S. cerevisiae* mutant strains.** The wild type (WT) and the mutants deficient for glycogen synthesis (gly^-^), neutral lipid synthesis (NL^-^) or both (NL^-^ gly^-^) were cultivated in C-lim minimal medium until glucose depletion and the survival rates in the following stationary phase were determined as described in Materials and Methods in the main text. No differences between the strains could be observed in the indicated time span.

**References**

DiCarlo JE, Norville JE, Mali P, Rios X, Aach J, Church GM. 2013. Genome engineering in *Saccharomyces cerevisiae* using CRISPR-Cas systems. *Nucleic Acids Res.* **41**:4336–43.

Petschnigg J, Wolinski H, Kolb D, Zellnig G, Kurat CF, Natter K, Kohlwein SD. 2009. Good Fat, Essential Cellular Requirements for Triacylglycerol Synthesis to Maintain Membrane Homeostasis in Yeast. *J. Biol. Chem.* **284**:30981–30993.
